# Mapping the national burden of cellulitis and related conditions in the United States, 1999–2024: a retrospective cohort analysis

**DOI:** 10.1097/MS9.0000000000005463

**Published:** 2026-08-06

**Authors:** Abdullah Altamimi, Abdulrahman Mohammed Dahi, Yazeed Sulaiman ALJohani, Wajal Alharbi, Abdulaziz Saud Alharbi, Abdulrahman M.H.M. Aldousari, Atheer G. Almutairi, Anas Ied Alenezi, Salah J.S.A.A. Alrashidi, Ahmed Ayed N Alanazi, Ahmed M. Alolyan, Laksh Kumar, Adarsh Raja, Aayush Chaulagain

**Affiliations:** a King Fahad Medical City, Riyadh, Saudi Arabia; b King Saud bin Abdulaziz University for Health Sciences, Jeddah, Saudi Arabia; c College of Medicine, King Abdulaziz University, Jeddah, Saudi Arabia; d College of Medicine, Qassim University, Buraydah, Saudi Arabia; e Department of Internal Medicine, Farwaniya Hospital, Ministry of Health, Al Farwaniyah, Kuwait; f College of Medicine, Northern Borders University, Arar, Saudi Arabia; g Department of Internal Medicine, Al-Sabah Medical Hospital, Ministry of Health, Kuwait City, Kuwait; h College of Medicine and Health Sciences, Arabian Gulf University, Manama, Bahrain; i Department of Internal Medicine, Shaheed Mohtarma Benazir Bhutto Medical College Lyari, Karachi, Pakistan; j Patan Academy of Health Sciences, Lalitpur, Nepal

**Keywords:** cellulitis, geographic disparities, mortality trends, public health surveillance, retrospective cohort analysis

## Abstract

**Background:**

Cellulitis is a common bacterial skin infection associated with substantial morbidity and economic burden in the United States, yet long-term national mortality trends remain poorly characterized.

**Methods:**

We analyzed multiple cause-of-death data from CDC WONDER from 1999 through 2024 using ICD-10 codes L03, H60, K12.2, and L98.3. Age-adjusted mortality rates (AAMRs) per 100 000 were calculated using the 2000 US standard population, with temporal trends assessed via Joinpoint regression across demographic, geographic, and urbanization subgroups.

**Results:**

A total of 139 485 cellulitis-related deaths were identified over the study period. The overall AAMR was 1.5 per 100 000, remaining stable from 1999 through 2011 before increasing significantly through 2024 [annual percent changes (APC): 2.39%, *P* = 0.0016]. Mortality rose sharply with age, with adults aged ≥85 years accounting for 31.0% of all deaths. Men had consistently higher AAMRs than women (1.7 vs. 1.4). NH American Indian or Alaska Native persons bore the greatest racial/ethnic burden (AAMR: 2.2), with rates rising from 2.2 in 1999 to 3.5 by 2024. The West and Midwest recorded the highest regional AAMRs (1.7 and 1.6, respectively), and nonmetropolitan areas sustained higher and more volatile mortality than metropolitan areas throughout (1.7 vs. 1.4).

**Conclusion:**

Cellulitis-related mortality increased significantly over recent decades and exhibits pronounced demographic and geographic disparities. Targeted prevention strategies, improved access to timely care for high-risk populations, and strengthened public health surveillance are needed to reduce this burden.

## Introduction

Over 14 million cases of cellulitis, one of the most prevalent bacterial skin infections, are reported in the United States each year, and the associated ambulatory care costs surpass $3.7 billion^[^1^]^. Additionally, the illness results in almost 650 000 hospital admissions annually, highlighting its significant healthcare burden. Clinically, Streptococcus pyogenes is the most common cause of cellulitis, which manifests as an acute infection of the deep dermis and subcutaneous tissue^[^2^]^. The main risk factor remains disruption of the skin barrier, which frequently results from traumatic injuries, surgical wounds, intravenous access sites, interdigital fissures, insect or animal bites, and coexisting skin conditions. People with peripheral arterial disease, diabetes mellitus, venous insufficiency, or lymphedema are especially at risk^[^3^]^.

HIGHLIGHTS

- National cellulitis mortality rose after 2011, reaching 139 485 deaths.
- Older adults, men, and American Indian/Alaska Native groups are most affected.
- The Midwest, the West, and nonmetropolitan areas showed higher and more volatile mortality.
- The findings highlight the urgent need for targeted prevention and better access to care.

Even though cellulitis is usually curable, serious side effects such as sepsis, bacteremia, and necrotizing fasciitis can cause significant morbidity and mortality, particularly in middle-aged and older adults. According to the current Infectious Diseases Society of America guidelines, patients with systemic signs of infection, hemodynamic instability, altered mental status, suspected deep infection, significant immunosuppression, poor treatment adherence, or failure of outpatient management should be hospitalized and receive parenteral antibiotic therapy^[^4–6^]^.

Despite cellulitis’s high prevalence and significant economic impact, little research has been done on its long-term mortality patterns. The majority of current research focuses on short-term results or clinical management, paying less attention to national trends and demographic differences in cellulitis-related deaths. Given the aging population and rising prevalence of comorbidities such as diabetes and vascular disease, it is essential to comprehend these patterns in order to inform prevention strategies and resource allocation. Thus, the purpose of this study is to examine national trends in cellulitis-related mortality in the United States between 1999 and 2024, with a focus on regional and demographic differences. No artificial intelligence (AI) tools were used in the research design, data collection, analysis, or interpretation, in compliance with the TITAN Guidelines 2025 for the transparent use of AI in scholarly communication; instead, AI support was restricted to language improvement during manuscript preparation^[^7^]^.

## Method

### Source of data and cohort definition

The Wide Ranging Online Data for Epidemiologic Research database of the Centers for Disease Control and Prevention (CDC WONDER) provided the death certificate records used in this study^[^8^]^. ICD10 diagnostic codes (L03, H60, K12.2, and L98.3) were used to identify deaths caused by cellulitis. The Multiple Causes of Death Public Use Registry contained death certificate data from all 50 states as well as the District of Columbia. Cases were categorized if cellulitis was identified as a contributing factor or as the primary cause of death. People were categorized according to age, from young children (younger than one year old) to senior citizens (older than 85 years old). Institutional review board approval was not necessary because the study only used publicly accessible, anonymized government data. This retrospective cohort study adhered to the Strengthening the Reporting of Cohort Studies in Surgery (STROCSS) guideline^[^9^]^.

### Data acquisition and classification

Population counts, year of death, place of death, urban/rural status, demographics, and state-level statistics were all included in the dataset. Hospices, private homes, long-term care facilities, hospitals, and nursing homes were among the places of death. Age, sex, race, and ethnicity were the demographic variables. Non-Hispanic (NH) Asian/Pacific Islander, NH Hispanic/Latino, NH American Indian/Alaska Native, NH Black/African American, and NH White were the categories. According to the National Center for Health Statistics, counties with fewer than 50 000 residents were classified as rural, while large metropolitan areas (≥1 million residents) and medium/small metropolitan areas (50 000–999 999 residents) were classified as urban^[^10^]^. The U.S. Census Bureau classified geographic regions into four categories: Northeast, Midwest, South, and West^[^11^]^.

### Statistical procedures

Age-adjusted mortality rates (AAMRs) and crude mortality rates (CMRs), expressed per 100 000 population with 95% confidence intervals (CIs), were used to assess mortality trends between 1999 and 2024. To facilitate comparisons across demographic groups and time periods, age adjustment was based on the 2000 US standard population^[^12^]^. Rates were examined by census region, state, race/ethnicity, sex, year, and urbanization status. Joinpoint regression (version 5.4.0, National Cancer Institute) was used to evaluate temporal changes and to identify statistically significant changes in mortality patterns^[^13^]^. When the *P*-value was less than 0.05 and the CI excluded zero, annual percent changes (APCs) and their corresponding 95% CIs were deemed significant. Microsoft Excel was used to create the figures.

## Results

A total of 139 485 cellulitis-related deaths were recorded during the study period. Females accounted for a slightly higher proportion of deaths than males, representing 51.8% (*n* = 72 182) compared with 48.2% among males (*n* = 67 303). Mortality increased markedly with advancing age, with adults aged ≥85 years constituting the greatest burden at 31.0% of all deaths, followed by those aged 75–84 years (25.6%) and those aged 65–74 years (19.2%). Younger age groups contributed minimally, with only 0.2% of deaths occurring among individuals aged 15–24 years (Table 1; Supplemental Digital Content Table S1, available at: https://links.lww.com/MS9/B336). Most deaths occurred in medical facilities (64.9%, *n* = 90 461), followed by nursing homes or long-term care facilities (16.1%, *n* = 22 481), and home settings (11.7%, *n* = 16 345) (Supplemental Digital Content Table S2, available at: https://links.lww.com/MS9/B336).

**Table 1:** Demographic Characteristics of Cellulitis-Related Mortalities in the United States, 1999–2024.

Variable	Deaths	AAMRs/CMRs (95% CI) per 100 000
Overall population^a^	139 485	1.5(1.5–1.5)
Sex^a^		
Male	67 303 (48.2%)	1.7(1.6–1.8)
Female	72 182 (51.8%)	1.4(1.3–1.4)
Census region^a^		
Northeast	23 565 (16.9%)	1.3 (1.2–1.4)
Midwest	33 800 (24.2%)	1.6 (1.5–1.7)
South	47 851 (34.3%)	1.4 (1.3–1.5)
West	34 269 (24.6%)	1.7 (1.6–1.8)
Race/ethnicity^a^		
American Indian or Alaska Native	1240 (0.9%)	2.2 (1.6–3)
Asian or Pacific Islander	2327 (1.7%)	0.6 (0.5–0.8)
Black or African American	10 753 (7.7%)	1.3(1.1–1.4)
White	116 015 (83.2%)	1.6 (1.6–1.7)
Hispanic or Latino	8471 (6.1%)	1.1 (0.9–1.2)
Urbanization^a^		
Metropolitan	111 880 (80.2%)	1.4(1.4–1.5)
Non-metropolitan	27 605 (19.8%)	1.7 (1.6–1.8)
Age groups^b^		
15–24 years	250 (0.2%)	
25–34 years	1281 (0.9%)	0.1 (0.1–0.1)
35–44 years	3752 (2.7%)	0.3 (0.3–0.3)
45–54 years	9601 (6.9%)	0.9 (0.9–0.9)
55–64 years	18 855 (13.5%)	2.0 (1.9–2.0)
65–74 years	26 752 (19.2%)	4.0 (4.0–4.1)
75–84 years	35 635 (25.6%)	9.6 (9.5–9.7)
85 + years	43 236 (31.0%)	29.9 (29.5–30.3)
Place of death^c^		
Medical facility	90 461 (64.9%)	–
Decedent’s home	16 345 (11.7%)	–
Hospice facility	6137 (4.4%)	–
Nursing home/long-term care facility	22 481 (16.1%)	–

^a^ Age adjusted mortality rates (AAMRs) are utilized.

^b^ Crude mortality rates (CMRs) are used for all age dependent analysis.

^c^ Age adjusted mortality rates (AAMRs) is not applicable for place of death.

### Overall mortality trends

**Figure 1.**
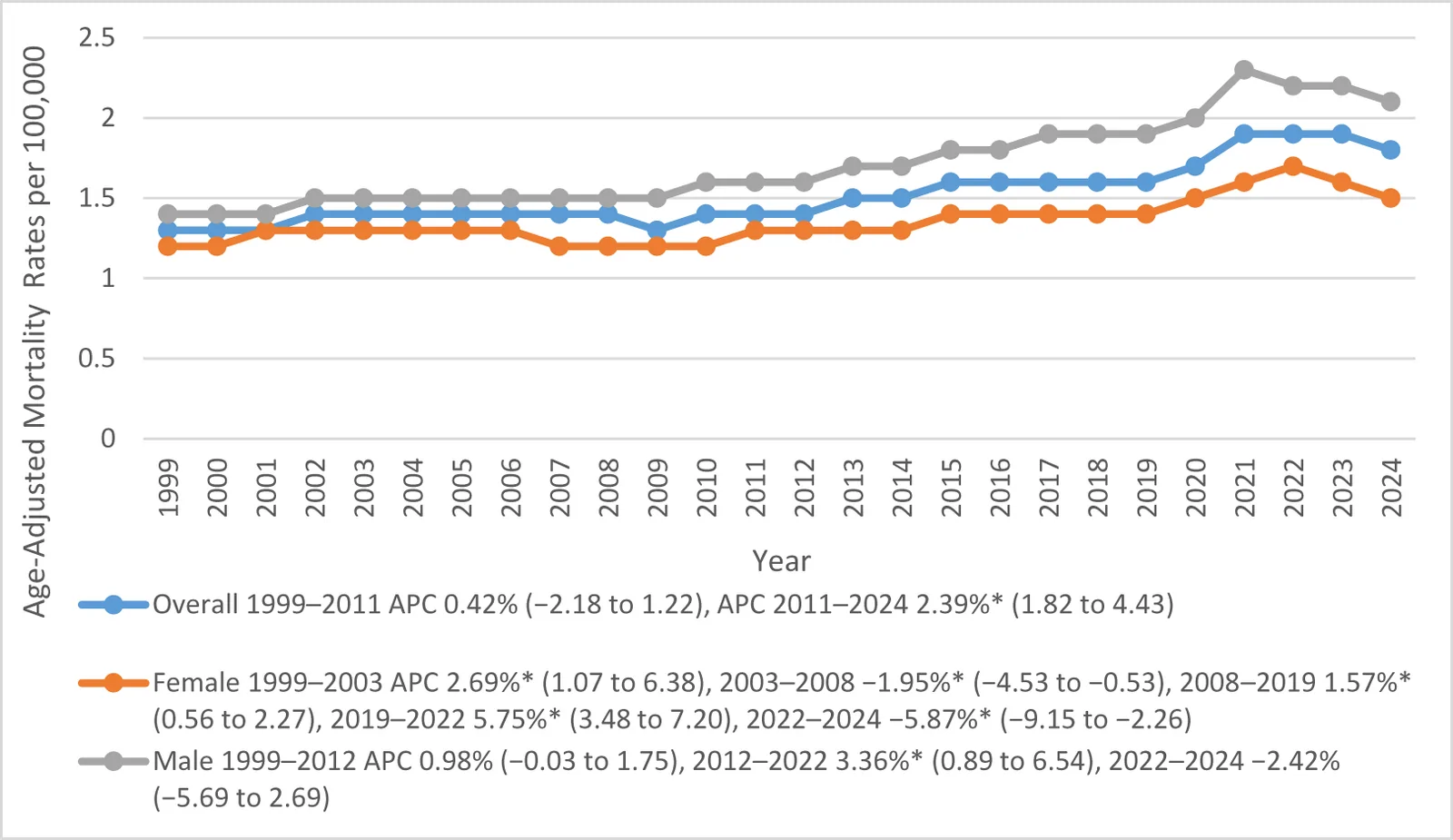
Age-adjusted mortality rates (AAMR) related to cellulitis from 1999 to 2024 in the United States, stratified by sex/gender.

Between 1999 and 2024, the overall AAMR for cellulitis-related deaths was 1.5 per 100 000 (95% CI: 1.5–1.5). Mortality remained stable from 1999 through 2011 (AAMR: 1.3 to 1.4; APC: 0.42%, *P* = 0.49), followed by a significant and sustained upward trend through 2024, when the AAMR reached 1.8 (APC: 2.39%, *P* = 0.0016). Temporal trends are illustrated in Fig. 1 and detailed in Supplemental Digital Content Tables S3–4, available at: https://links.lww.com/MS9/B336.

### Mortality trends by sex

Throughout the study period, men sustained a consistently higher AAMR than women (1.7 vs. 1.4 per 100 000). Among women, mortality followed a fluctuating trajectory, rising modestly through 2003, declining slightly through 2008, increasing again through 2019, and accelerating sharply between 2019 and 2022 (the steepest rise observed in this group) before declining through 2024. Among men, mortality showed a more sustained upward pattern, with a marked and significant acceleration between 2012 and 2022 (AAMR: 1.6 to 2.2), followed by a modest, non-significant decline through 2024 (AAMR: 2.1). Full segment-level APC estimates for both sexes are provided in Supplemental Digital Content Tables S3–4, available at: https://links.lww.com/MS9/B336.

### Mortality trends by race/ethnicity

**Figure 2.**
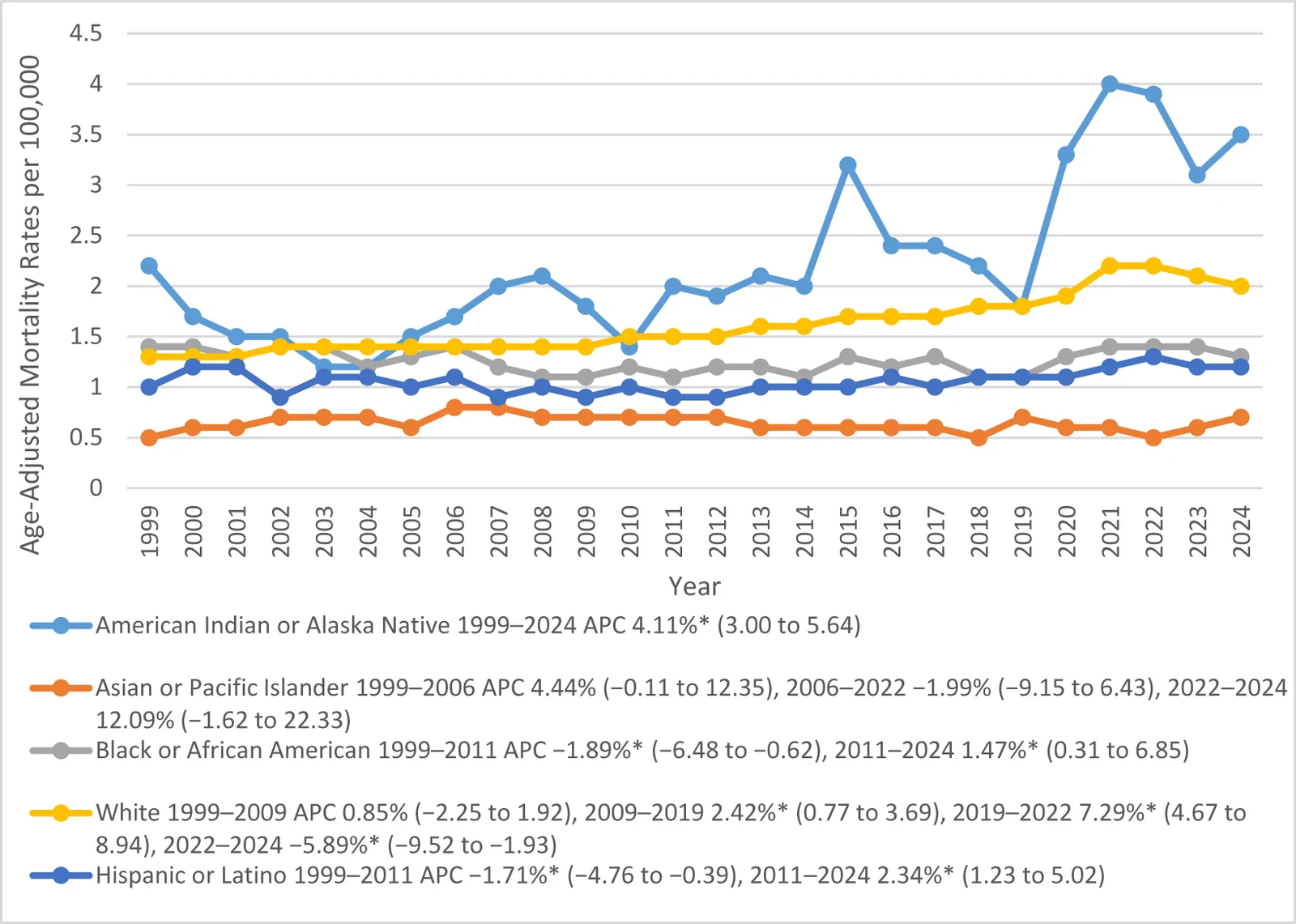
AAMR related to cellulitis from 1999 to 2024 in the United States, stratified by race/ethnicity.

Cumulative AAMRs differed substantially across racial and ethnic groups, with the highest rates among NH American Indian or Alaska Native persons (2.2, 95% CI: 1.6–3.0), followed by NH White (1.6, 95% CI: 1.6–1.7), NH Black or African American (1.3, 95% CI: 1.1–1.4), Hispanic or Latino (1.1, 95% CI: 0.9–1.2), and NH Asian or Pacific Islander persons (0.6, 95% CI: 0.5–0.8). NH American Indian or Alaska Native populations showed the most pronounced and sustained increase across the entire study period, with the AAMR rising from 2.2 in 1999 to 3.5 in 2024 (APC: 4.11%, *P* < 0.000001). NH White persons experienced the steepest short-term acceleration between 2019 and 2022 (APC: 7.29%, *P* = 0.0004), followed by a significant decline through 2024. NH Black persons showed an initial significant decline through 2011, followed by a significant reversal through 2024. Hispanic persons followed a similar biphasic pattern of early decline and subsequent increase. Trends among NH Asian or Pacific Islander persons did not reach statistical significance in any segment. Race/ethnicity-specific trends are illustrated in Fig. 2 and detailed in Supplemental Digital Content Tables S3–5, available at: https://links.lww.com/MS9/B336.

### Mortality trends by census region

**Figure 3.**
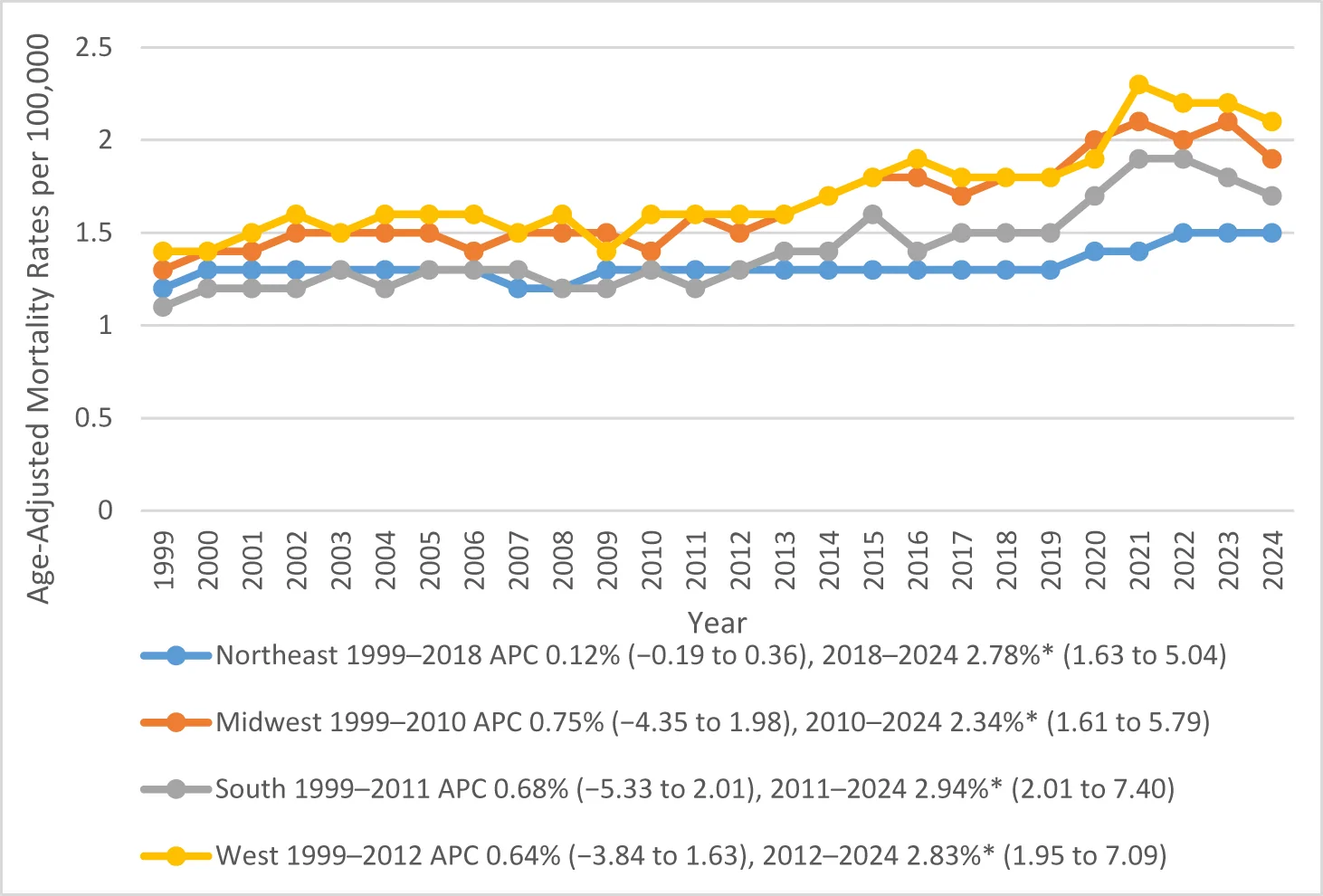
AAMR related to cellulitis from 1999 to 2024 in the United States, stratified by census region.

Regional AAMRs were highest in the West (1.7; 95% CI, 1.6–1.8) and the Midwest (1.6; 95% CI, 1.5–1.7), and lowest in the Northeast (1.3; 95% CI, 1.2–1.4). All four regions shared a common pattern of relative stability during the earlier part of the study period, followed by a significant upward trend emerging between the late 2000s and the early 2010s and persisting through 2024. The South showed the latest inflection point, with a significant increase beginning in 2011 (APC: 2.94%, *P* = 0.027), while the Northeast showed the most recent shift, with a significant rise beginning only in 2018 (APC: 2.78%, *P* < 0.000001). Regional trends are illustrated in Fig. 3 and detailed in Supplemental Digital Content, Tables S3–6, available at: https://links.lww.com/MS9/B336.

### Mortality trends by urbanization

**Figure 4.**
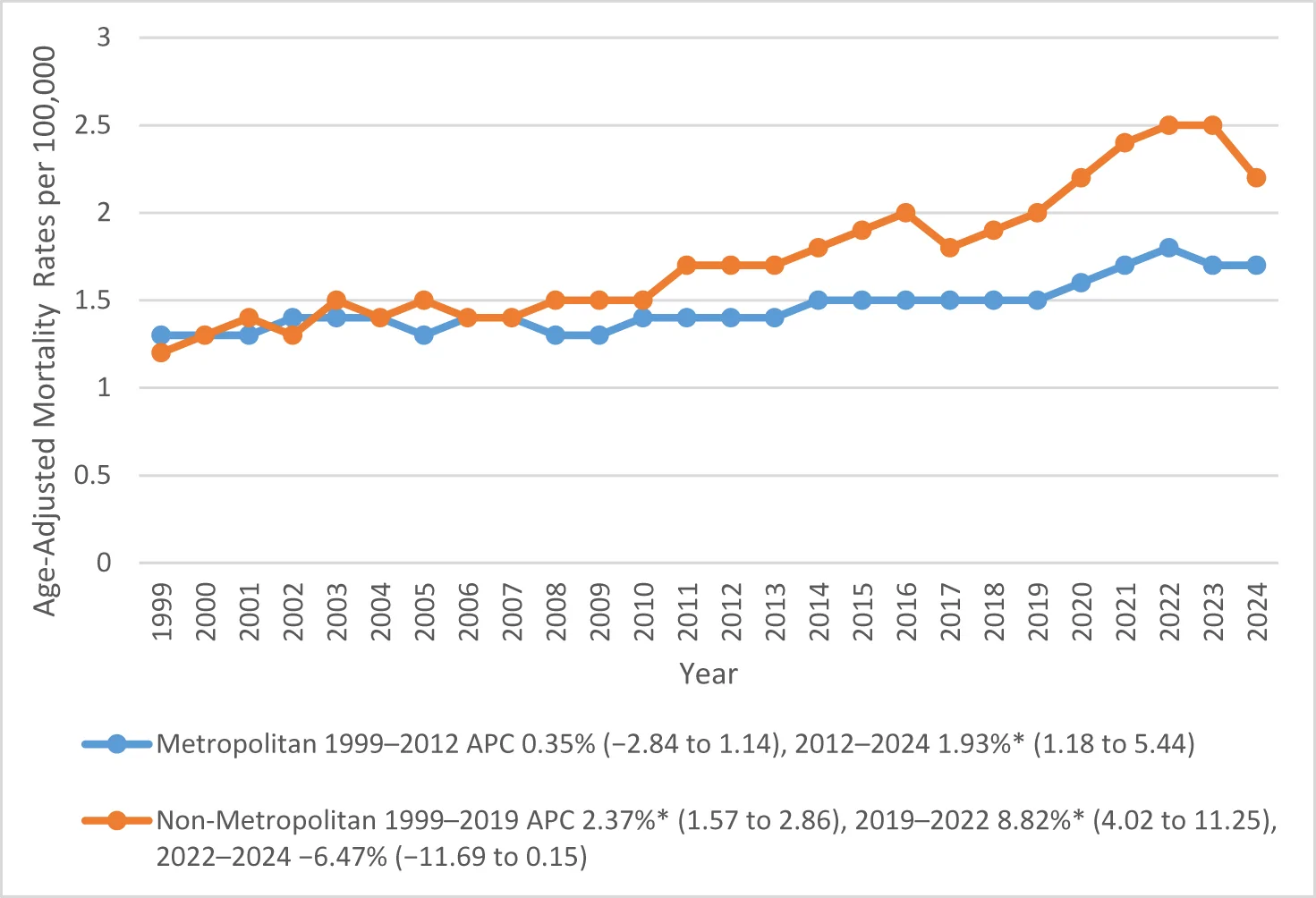
AAMR related to cellulitis mortality from 1999 to 2024 in the United States, stratified by urbanization.

Nonmetropolitan areas sustained a consistently higher AAMR than metropolitan areas throughout the study period (1.7 vs. 1.4 per 100 000). Metropolitan mortality remained stable through 2012 before rising significantly through 2024 (APC: 1.93%, *P* = 0.026). In contrast, nonmetropolitan areas showed a sustained significant increase from 1999 through 2019, followed by a sharp acceleration between 2019 and 2022 (AAMR: 2.0 to 2.5; APC: 8.82%, *P* = 0.011)—the most pronounced surge observed across any subgroup–before a modest, nonsignificant decline through 2024. Urbanization-specific trends are illustrated in Fig. 4 and detailed in Supplemental Digital Content Tables S3–7, available at: https://links.lww.com/MS9/B336.

### State-level distribution

**Figure 5.**
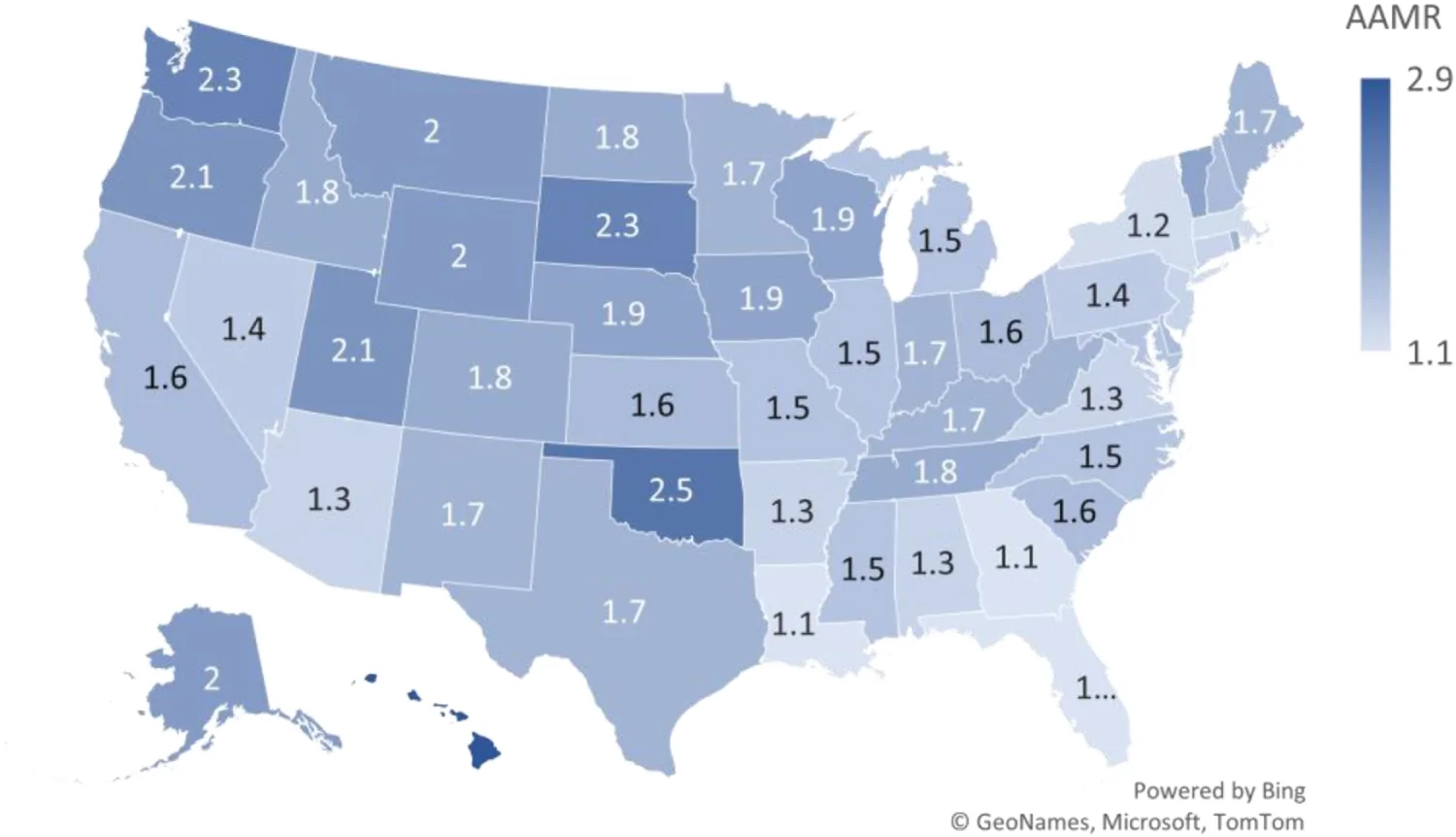
AAMR related to cellulitis from 1999 to 2024 in the United States, stratified by state.

Substantial state-level variation was observed. The highest AAMRs were concentrated in the Pacific and Mountain West, with Hawaii recording the greatest burden (2.9, 95% CI: 2.6–3.1), followed by Oklahoma (2.5), South Dakota (2.3), Washington (2.3), and Oregon (2.1). The lowest AAMRs were observed in the Southeast and Northeast, with Florida, Georgia, and Louisiana each recording 1.1, and Massachusetts and New York each recording 1.2. This distribution reflects a nearly threefold disparity between states with the highest and lowest burdens (Fig. 5; Supplemental Digital Content Table S8, available at: https://links.lww.com/MS9/B336).

## Discussion

**Figure 6.**
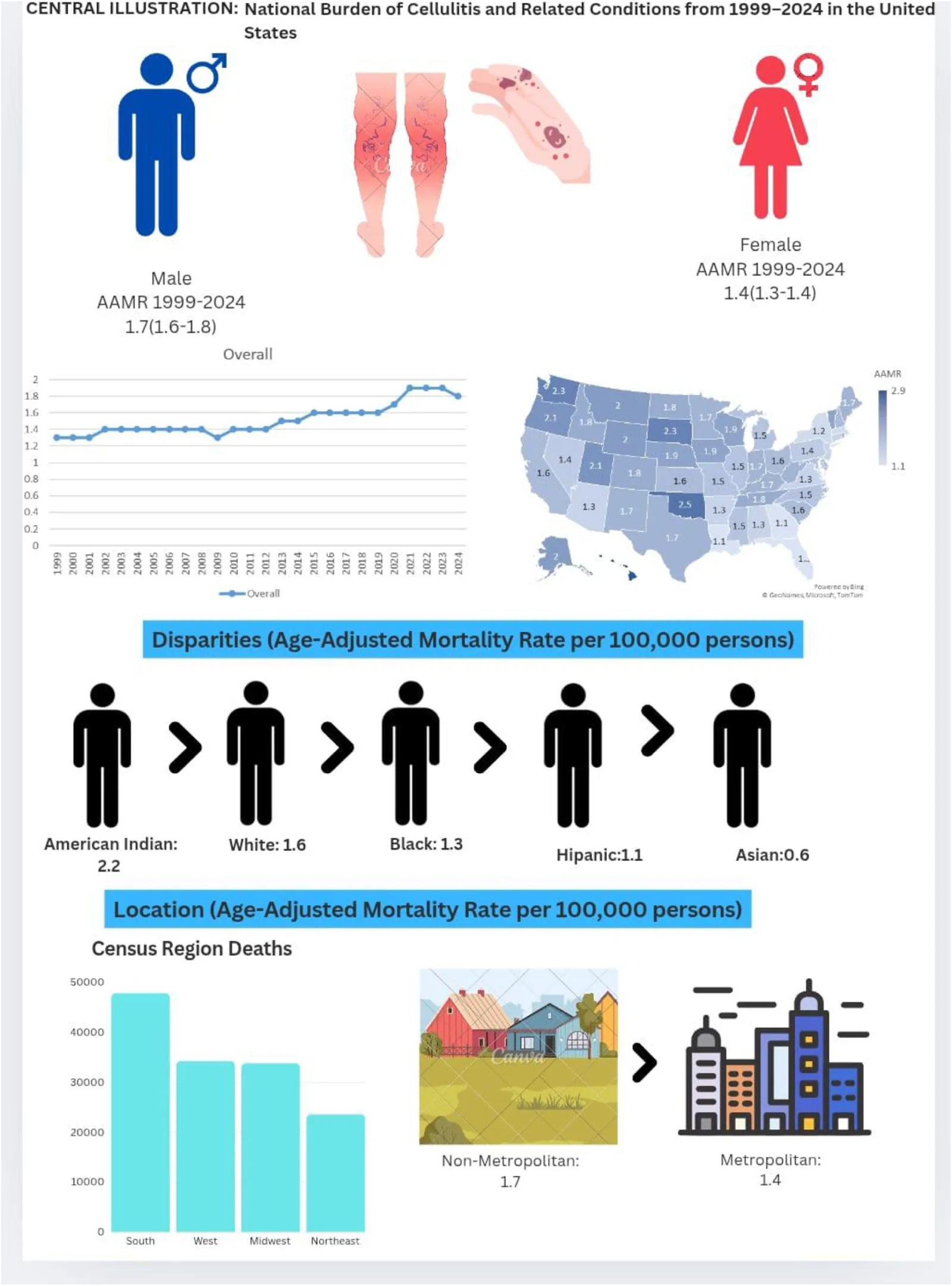
Demographic profiles and disparities in mortality related to cellulitis from 1999 to 2024 in the United States.

According to this national analysis, cellulitis is still a significant and rising cause of death in the United States. Older adults accounted for the majority of deaths during the study period, highlighting the increased susceptibility of aging populations. There were evident demographic disparities, with men bearing a higher mortality burden than women and the highest rates among racial and ethnic groups occurring among American Indians and Alaska Natives. Geographic differences were also apparent, with some Western states bearing a noticeably heavier burden than those in the Southeast and Northeast, and rural communities consistently experiencing higher mortality than metropolitan areas. Taken as a whole, these trends show growing regional and demographic disparities and emphasize the necessity of focused prevention and better access to care for high-risk groups (Fig. 6).

Due to physiological and systemic vulnerabilities, cellulitis mortality exhibits a clear age gradient, with older adults having much higher mortality rates. A study found that patients over 65 had a 9.37-fold increased mortality risk, providing compelling evidence for this claim^[^14^]^. According to a different study, comorbid conditions such as dementia, atrial fibrillation, and hypertension were more common in older patients (75+) and were associated with an increased risk of death^[^15^]^. The cumulative health vulnerabilities are reflected in the age-related mortality gradient, in which older adults have more complicated clinical presentations and are more prone to complications.

Despite the extremely small amount of prior research on gender differences in cellulitis, the patterns found in the few studies that are currently available do not fully match the mortality trends found in our analysis. According to one study, women are more likely than men to develop cellulitis at a later age. This suggests that age alone may be a risk factor for cellulitis in women, independent of comorbidities^[^16^]^. Nevertheless, the results of that study were derived from small cohorts with limited generalizability, and it concentrated on clinical presentation rather than mortality. On the other hand, our national assessment shows that men have a higher overall mortality burden, even though women present later in life in other settings. This disparity implies that, depending on whether incidence, disease severity, or mortality is being assessed, gender-related variations in cellulitis outcomes may differ. The more severe outcomes we saw are probably caused by factors such as differences in occupational exposures among men, higher rates of comorbid vascular disease, and delayed healthcare-seeking behavior^[^17,18^]^. Therefore, our results show that men are still at higher risk of fatal cellulitis, despite previous literature highlighting possible age-related vulnerabilities among women. This highlights the need for more research that is specifically focused on gender-based differences in disease severity and outcomes.

American Indian/Alaska Native populations face the greatest health challenges, and racial and ethnic mortality disparities reveal deep and systemic health inequities throughout the United States. The evidence is solid and consistent with other research. American Indian/Alaska Native populations had the highest all-cause mortality rate in 2019 (1028.2 per 100 000), significantly higher than that of White populations (802.5 per 100 000), according to a 2023 study^[^19^]^. These differences were corroborated by another study, which found that rural counties with a majority American Indian/Alaska Native population had noticeably higher premature death rates^[^20^]^.

Previous research has demonstrated that Black patients are more likely to be hospitalized for cellulitis; this trend is frequently linked to delayed access to outpatient care and a lack of preventive dermatologic care^[^21^]^. These findings are consistent with evidence that certain minority populations are more likely to have comorbid conditions like diabetes, peripheral vascular disease, and chronic edema, which increase susceptibility to severe cellulitis^[^22^]^. More advanced clinical presentations among racial and ethnic minorities are also caused by obstacles to prompt evaluation by primary care, dermatology, and infectious disease specialists. Despite the generally low mortality rate associated with cellulitis, persistent outcome gaps are reinforced by disparities in baseline health status and unequal access to early intervention, which mirror broader patterns seen across multiple infectious conditions in the United States^[^19^]^. Within this context, the disparities identified in our study likely reflect the intersection of underlying comorbidity profiles and structural inequities that influence opportunities for early recognition, treatment, and follow-up.

Significant regional differences in cellulitis mortality highlight how environmental, structural, and healthcare system factors affect results. The West and Midwest had higher mortality rates overall, with a number of Western states having the highest burden^[^22^]^. The capacity of the health system, the availability of inpatient and outpatient infectious disease services, the prevalence of chronic comorbidities, and underlying socioeconomic conditions could all be factors in these patterns^[^23^]^. Delays in diagnosis and treatment frequently result in worse outcomes in areas with higher rates of poverty, fewer healthcare facilities, and a lower density of clinicians. States in the Southeast and Northeast, on the other hand, showed relatively lower mortality rates, which may have been due to better healthcare facilities, greater clinician availability, and more developed procedures for treating acute skin infections^[^24^]^.

The absence of clinical covariates in death certificate data has important implications for interpreting the observed mortality patterns^[^25^]^. Comorbidities such as diabetes mellitus, peripheral vascular disease, chronic venous insufficiency, and obesity are well-established risk factors for severe cellulitis and fatal outcomes, yet their distribution across demographic and geographic subgroups cannot be assessed within this dataset. Similarly, infection severity at presentation, microbiological etiology, antibiotic selection, and treatment adequacy, all of which independently influence prognosis, are unavailable. As a consequence, the demographic and geographic disparities identified in this study may reflect differences in underlying comorbidity burden, healthcare access, or disease severity rather than mortality risk attributable to cellulitis per se. This limits the internal validity of subgroup comparisons and precludes causal attribution. The findings should therefore be interpreted as population-level descriptive patterns that identify high-burden groups warranting targeted investigation, rather than as evidence of independent mortality risk.

Disparities between urban and rural areas highlight structural injustices that influence mortality risk. There are frequently fewer hospitals, dermatologists, and infectious disease specialists in rural areas. Due to this limited access, diagnosis and treatment are delayed, which raises the possibility that cellulitis will worsen and result in necrotizing fasciitis or sepsis. In general, outcomes are improved in urban areas due to higher clinician density, more specialized care, and established pathways for managing acute infections. When cellulitis develops into a systemic infection, the intensive care capacity of rural hospitals is frequently limited, which can worsen outcomes. Survival rates are higher in urban hospitals because they are more likely to have advanced diagnostic support and infectious disease units^[^26–28^]^.

## Limitations

The use of CDC WONDER data, which may contain coding errors, underreporting, or incorrect classification of cellulitis-related deaths, limits this study. The absence of individual-level clinical data represents the most consequential limitation of this study. CDC WONDER death certificate data do not capture comorbidity profiles, infection severity at presentation, microbiological etiology, antibiotic regimens, or treatment outcomes. Because these variables are distributed unevenly across demographic and geographic subgroups, residual confounding is likely present in all stratified analyses. For example, the higher mortality burden observed among American Indian/Alaska Native populations and rural communities may partly or substantially reflect differences in the prevalence of predisposing comorbidities such as diabetes and peripheral vascular disease or differences in access to timely care, rather than an independent effect of demographic or geographic factors alone. This limits the internal validity of subgroup comparisons and means that no causal conclusions can be drawn from the observed associations. Furthermore, even though we found regional and demographic disparities, the data do not include social determinants of health, healthcare-seeking behavior, or access to care, all of which probably contribute to the observed differences.

## Conclusion

Men, older adults, and racial and ethnic minorities, especially American Indian and Alaska Native populations, are disproportionately affected by cellulitis, which continues to be a major and increasing cause of death in the United States. The impact of infrastructure, structural injustices, and healthcare access on outcomes is highlighted by geographic and urban-rural disparities. To lower mortality and address enduring demographic and regional disparities, these findings highlight the necessity of targeted prevention strategies, early detection, and equitable access to care, particularly for high-risk populations.

## Data Availability

All data used in this study are publicly accessible through the CDC WONDER database (https://wonder.cdc.gov). No individual-level or identifiable patient data were used.

## Research registration unique identifying number (UIN)

AMSU-D-25-03876.
